# Unveiling the pH-Responsive Mechanisms of the Carbon Dot–Proximicin-A Peptide Conjugate for Targeted Cancer Therapy Using Density Functional Theory

**DOI:** 10.3390/molecules30040896

**Published:** 2025-02-14

**Authors:** Azeez Ahamed, Piumantha Samaranayake, Visal de Silva, Muhammad Raziq Rahimi Kooh, Nadeesha Wickramage, Indu G. Rajapaksha, Roshan Thotagamuge

**Affiliations:** 1 Department of Physics, Faculty of Science, University of Ruhuna, Matara 81000, Sri Lanka; azeezahamed1997@gmail.com (A.A.); samaranayakepiumantha@gmail.com (P.S.); desilvavisal@gmail.com (V.d.S.); 2 Centre for Advanced Material and Energy Sciences, Universiti Brunei Darussalam, Jalan Tungku Link, Gadong BE1410, Brunei; raziq.kooh@ubd.edu.bn; 3 Department of Basic Sciences, Faculty of Allied Health Sciences, University of Sri Jayewardenepura, Nugegoda 10250, Sri Lanka; indurajapaksha@sjp.ac.lk; 4 Department of Optometry, Faculty of Allied Health Sciences, University of Sri Jayewardenepura, Nugegoda 10250, Sri Lanka

**Keywords:** carbon dots and proximicin-A peptide, density functional theory, targeted drug delivery

## Abstract

This study investigates the pH-responsive dissociation mechanism of carbon dot (CD) conjugated with the anticancer peptide proximicin-A (PROXI) using density functional theory (DFT) simulations. The CD@PROXI system, designed for targeted cancer therapy, releases the drug in acidic environments typical of cancer sites. DFT simulations, with the B3LYP-D3BJ functional and 6-311G (d, p) basis set, optimized the conjugate’s geometry under neutral and acidic conditions. The focus was on the pH-sensitive C=N bond, existing in two protonation states. Key parameters evaluated included the HOMO-LUMO gap, bond length, IR spectroscopy, non-covalent interaction (NCI), electron localization function (ELF), density of states (DOSs), and electrostatic potential (ESP). Under neutral pH, the system showed stability with a HOMO-LUMO gap of 3.22 eV, indicating low reactivity. In acidic pH, this gap decreased to 0.40 eV, suggesting higher reactivity and potential for drug release. IR spectroscopy indicated weakened C=N bonds in acidic conditions, with bond length increasing from 1.288 Å to 1.324 Å. NCI analysis revealed increased van der Waals interactions, supporting bond weakening. ELF analysis showed electron localization at reactive sites, while DOS profiles and ESP maps highlighted distinct electronic states and potential dissociation regions in acidic conditions. These findings confirm the potential of CD@PROXI for targeted cancer therapy, with drug release triggered by the acidic tumor microenvironment.

## 1. Introduction

Globally, cancer is responsible for one in every six deaths [1], marking it as a major health challenge. Current treatment options include chemotherapy, surgery, radiation therapy, or a combination thereof. However, these methods lack specificity, often affecting both cancerous and healthy cells, leading to various adverse side effects such as nausea, vomiting, hair loss, bruising, bleeding, anemia, and infertility [2]. Consequently, there is a critical need for innovative treatments. Recent advancements include stem cell therapy [3], ablation therapy [4], and targeted drug delivery systems [5].

Stem cell therapy involves using stem cells. These are specialized cells capable of differentiating into various cell types, primarily sourced from bone marrow, peripheral blood, or umbilical cord. Hematopoietic stem cell transplantation (HSCT) is one application of this therapy, aimed at replacing damaged or malignant bone marrow to treat blood cancers like leukemia, lymphoma, and multiple myeloma [3]. Beyond oncology, stem cell therapy shows promise in treating neurodegenerative diseases such as Parkinson’s disease, multiple sclerosis, amyotrophic lateral sclerosis, and spinal cord injuries [6]. Ablation therapy targets and destroys malignant tissue with minimal damage to surrounding healthy tissues. Techniques include microwave ablation, which uses microwave radiation to heat and kill cancer cells; radiofrequency ablation (RFA), employing high-frequency electrical currents; and cryoablation, which involves freezing cancer cells to death. These methods are often applied to small malignant renal masses [7].

Targeted cancer therapy utilizes molecular targeting agents, such as monoclonal antibodies and small molecule inhibitors, to interfere with cancer cell growth. This approach typically involves a drug and a drug carrier, conjugated to form a drug delivery system. The carrier facilitates targeted delivery to cancer cells, exploiting the acidic environment of cancerous tissues, where the pH ranges from 6.2 to 6.9 compared to the pH range of 7.0 to 7.2 in healthy cells [8]. This pH sensitivity is harnessed to design drug systems that are stable in the alkaline environment of healthy cells but release their payload in the acidic milieu of cancer cells.

Carbon dots, nanoparticles with versatile functional properties, have recently attracted significant attention as effective drug carriers in cancer therapy [9]. Their combination with anticancer peptides presents a promising strategy for targeted drug delivery systems. Anticancer peptides (ACPs) are small chains of amino acids, typically ranging from 10 to 60 in length, with the ability to prevent tumor cell growth, block their spread, or reduce blood vessel formation within tumors [10]. Carbon dots are favored for their non-toxicity, excellent water dispersion, and pH-responsive drug release properties [11,12], Anticancer peptides, which can be modified to be positively charged and form alpha-helical structures, interact with the negatively charged cancer cell surfaces, facilitating penetration and inducing apoptosis through the mitochondrial release of cytochrome C [13].

Despite numerous experimental studies, there is a lack of comprehensive simulation studies on the mechanistic aspects of loading and releasing anticancer peptides using carbon dots. This study aims to address this gap by employing density functional theory (DFT) simulations to explore the stability, electronic structure, and energetic attributes of carbon dots conjugated with anticancer peptides under low pH conditions. For instance, a study has employed DFT to investigate graphyne as a drug carrier for daunorubicin. By analyzing adsorption energy, charge transfer, and non-covalent interactions, it demonstrated that graphyne allows effective daunorubicin adsorption and pH-dependent desorption through weak van der Waals interactions [14]. In contrast, our study utilizes carbon dots as a drug carrier for an anticancer peptide, leveraging their unique chemical and electronic properties for targeted drug delivery.

DFT is a powerful quantum mechanical framework that provides insights into the electronic structure and properties of atoms and molecules by analyzing electron density. This approach circumvents the complexities of solving the many-electron Schrödinger equation [15]. For this study, Gaussian software was utilized to perform geometry optimization, frequency analysis, and energy calculations.

A carbon dot (CD) with three linearly aligned graphene rings was selected for its balance of complexity and adequacy to the model [16]. Its functionalization with a hydrazine group introduces a facility to form a pH-sensitive C=N bond with the anticancer peptide, which is crucial for the pH-induced drug release [17]. On the other hand, proximicin-A peptide (PROXI) was chosen as the anticancer peptide due to its relatively simple structure and its ability to target cancer cells such as human gastric adenocarcinoma (AGS), human hepatocellular carcinoma (HepG2), and human breast carcinoma (MCF 7) [18]. PROXI, similar in structure to pyrrolamidone antibiotics, exhibits antitumor activity through selective binding to AT-rich DNA sequences, inducing cell cycle arrest. This prevents cancer cells from advancing to DNA replication and division [19,20]. Other in vitro studies revealed that PROXI binds well to receptors linked to cancer progression, such as HER2, a protein implicated in aggressive breast cancer. PROXI has been shown to suppress the growth of cancer cells and promote cell death in models such as glioblastoma and breast cancer, suggesting its potential as a targeted treatment for these cancers [21]. The conjugation of PROXI with the carbon dot involves a covalent bond between the N atom of the hydrazine group and the C atom of the carbonyl group in PROXI. This bond is pH-sensitive and protonates in acidic environments [17]. Several in vivo studies have ensured the biocompatibility and low toxicity of CDs. For instance, one study pointed out that CDs injected into cancer-bearing mice completely cleared from their bodies within 24 h indicating excellent biocompatibility of CDs [22]. CDs also showed low retention in organs such as the spleen and liver, indicating their less likelihood to accumulate in major organs, while they showed preferential accumulation in tumor sites [23]. These studies highlight the potential of CDs as drug carriers in targeted drug delivery systems while minimizing off-target effects. In an experiment, CD was studied as a drug carrier where it was conjugated with doxorubicin (DOX) to form a targeted drug system CD@DOX. The system demonstrated effective localization at tumor sites, reducing systemic toxicity compared to free DOX. In comparison, the CD@PROXI system, where DOX is replaced with PROXI, will demonstrate a similar behavior for pH-induced targeted drug delivery, minimizing off-target effects [24]. The collection of these experimental and in vivo studies suggests the excellent biocompatibility and low toxicity of CDs as drug carriers and the precision of CD@PROXI in targeting cancer sites, which ensures minimal off-target effects.

The objective of this study was to investigate how the conjugation of CD with PROXI (CD@PROXI) dissociates in acidic pH conditions, which we observe in a cancer cell compared to a normal cell. DFT simulations were employed to analyze the stability, electronic structure, and pH-responsive behavior of the drug delivery system, providing insights into its potential efficacy in targeting cancer cells.

## 2. Results and Discussion

### 2.1. CD Analysis

The structure of CDs includes various orientations of carbon rings, such as linear chains, triangles, diagonals, and parallelograms. Based on the UV-Vis spectra, the aromatic rings in linear chain structures exhibit absorption bands similar to those obtained in experimental measurements, indicating π-π* transitions. Therefore, the chain structures of CDs are suitable for modeling the behavior of CDs [16]. Considering these factors, the choice of CD structure in this study was a linear chain with three carbon rings. Generally, CDs can have functional groups containing oxygen and nitrogen atoms on their surface. In this research, a hydrazine group containing a nitrogen atom was bonded to the surface of the CD. When the CD functionalized with the hydrazine group was bonded with the anticancer peptide, the hydrazone linkage formed between the anticancer peptide PROXI and its carrier CD, which offers a promising approach for pH-responsive drug delivery by enabling targeted release in acidic conditions. Due to their accelerated breakdown in lower pH environments, hydrazone linkages within CD@PROXI can support the controlled release of PROXI specifically at acidic sites, such as tumor regions or inflamed tissues. This pH-sensitive property enhances the release of the anticancer agent where it is most needed, potentially reducing systemic side effects and improving therapeutic effectiveness at the target location [17].

The linear chain structure of the CD as a drug carrier offers the advantage of a higher surface area-to-volume ratio, allowing for more efficient drug loading and delivery. Although in this study only one functional group is considered for simplicity, where the complexity of the system is reduced to focus on the basic principles of drug loading and delivery, the linear chain structure inherently provides greater flexibility to accommodate additional surface functional groups. This expanded functionalization capability enables the loading and delivery of multiple drugs simultaneously, potentially allowing for a more targeted and synergistic therapeutic effect at specific sites, such as cancer cells or inflamed tissues. Consequently, the linear chain structure not only enhances drug-carrying capacity but also opens up possibilities for combination therapies, increasing the efficacy of the drug delivery system [25].

**Figure 1:**
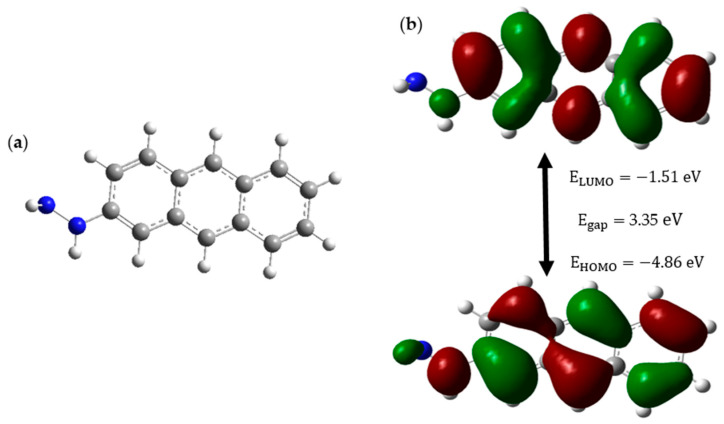
(**a**) Optimized CD structure (Blue, white, and grey represent Nitrogen, Hydrogen, and Carbon atoms respectively) and (**b**) HOMO-LUMO structure of CD.

The CD structure is subjected to HOMO-LUMO analysis to obtain insights into its stability and availability as a drug carrier in pH-controlled drug release. Below, Figure 1 shows the CD structure and its relevant HOMO-LUMO orbitals and energy profile of the CD.

Figure 1a illustrates the structure of the CD, while Figure 1b reveals that the CD has a low HOMO energy of −4.86 eV and a high-energy gap of 3.35 eV, which gives it the attributes of a stable and less reactive molecule. The surface functionalization of the hydrazine group (NH_2_-NH-) to the CD makes sure that this drug carrier is soluble in water because it contains a hydrophilic NH_2_ group. Furthermore, the NH_2_ fragment of hydrazine possesses the ability to form an N=C bond with the anticancer drug, where this bond will act as the bridge between the drug carrier and the anticancer drug, and this bond is extremely sensitive to acidic conditions [26]. This sensitivity is such that the bond tends to break even in the mild acidic environment of cancer sites of pH ranging between 6.4 and 7.0 [27]. Hence, from the HOMO-LUMO analysis, it is clear that our CD has properties including high stability and low reactivity. These two properties ensure that our CD will remain stable and inert inside the patient despite the altering of the cellular environment from neutral to acidic. Surface functionalization of CD with hydrazine gives additional properties like high solubility, which facilitates easy movements within body fluids, and pH-responsive drug release, which is the most important aspect of this study. Hence, we suggest that the linear chain structure of CD with three carbon rings, which was functionalized with hydrazine, is an excellent choice as a drug carrier to carry anticancer drugs precisely to cancer sites.

### 2.2. PROXI Analysis

Proximicins are aminofuran antibiotics extracted from marine members of the rare genus *Verrucosispora* strain MG-37. Later studies revealed that PROXI exhibits anticancer properties that can inhibit the growth of cell lines such as gastric adenocarcinoma, hepatocellular carcinoma, and breast carcinoma [18]. Therefore, the selection of PROXI gives the advantage of its dual functionality, where it can be used for cancer treatment as well as post-treatment infections in cancer patients.

For PROXI to qualify as an anticancer peptide drug in the targeted treatment of cancer, in addition to the above-mentioned qualities, it should also possess two qualities, which include the ability to dissociate its bond with the carrier, specifically at cancer sites, and the ability to be attracted towards cancer cells after the bond dissociation. PROXI was investigated to find whether the above two qualities are present or not using DFT results.

**Figure 2:**
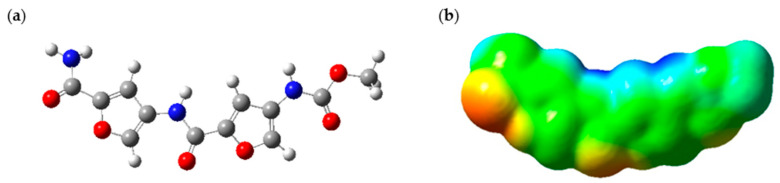
(**a**) Optimized PROXI structure (Blue, white, grey and red represent Nitrogen, Hydrogen, Carbon and Oxygen atoms respectively) and (**b**) ESP map of PROXI.

The PROXI structure has several C=O groups where the NH_2_ group of hydrazine could react with C=O groups to form a pH-sensitive C=N bond. Therefore, it is evident that PROXI has the potential to break its bond with the drug carrier selectively at acidic cancer cell sites. Hence, the first quality that PROXI should possess, which is the ability to dissociate its bond with the carrier specifically at cancer sites, is met. Suppose that the bond was dissociated near a cancer site and PROXI has been released. Now it is important to investigate whether PROXI would satisfy the second condition, which is the ability to be attracted towards cancer cells. To obtain insights about this quality, an ESP map of released PROXI was studied. Figure 2 shows the illustration of the optimized PROXI molecule and its relevant ESP map. ESP maps provide valuable insights into the electrostatic density variation in a molecule. These colored maps range from red to blue, where the red color indicates regions of minimum electrostatic potential and blue regions indicate the opposite. Green regions indicate the neutral ESP regions of the molecule [28].

The ESP map of the optimized PROXI structure in Figure 2a was demonstrated in Figure 2b. ESP map provides significant insights into its potential interactions with cancer cells. The map predominantly features neutral regions, indicated in green, where van der Waals forces can facilitate interactions with neighboring molecules. Additionally, the ESP map reveals significant red areas, representing regions of negative electrostatic potential (electron-rich regions), which likely act as nucleophilic sites. Conversely, the blue regions indicate areas of positive electrostatic potential (electron-deficient regions), crucial for electrostatic attraction to the negatively charged surfaces of cancer cells. Therefore, a released PROXI at a cancer cell is expected to interact electrostatically with the nucleophilic negatively charged components of cancer cells through these blue regions and draw nearer to the cancer cell. Meanwhile, the extensive green regions enhance these interactions by supporting van der Waals forces, promoting overall molecular binding and stability.

**Figure 3:**
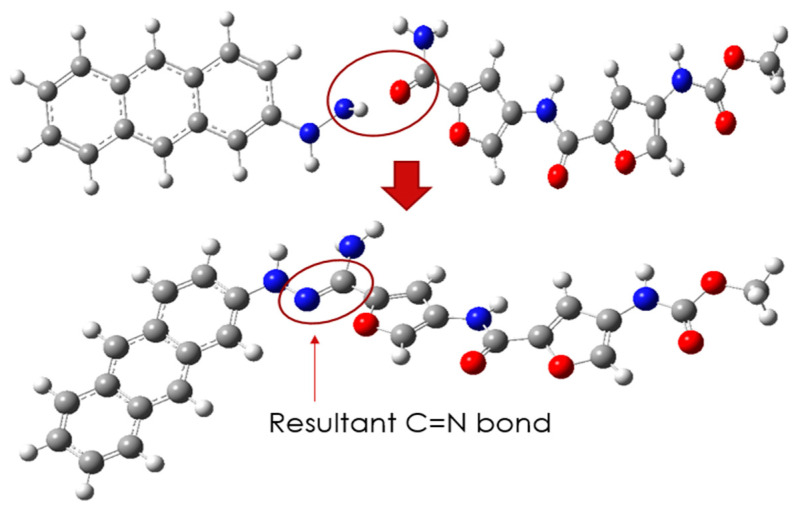
Formation of CD@PROXI by conjugating CD and PROXI via N=C pH-sensitive bond.

Since it is unambiguous that CD and PROXI are well-suited candidates as the drug carrier and the drug, the targeted drug system can be made by conjugating them via a pH-sensitive N=C bond. Figure 3 shows the process of conjugation of CD and PROXI and the resultant drug system CD@PROXI.

The newly formed CD@PROXI, as shown in Figure 3, serves as a model for a targeted treatment drug system for cancer. However, it would be premature to conclude its effectiveness solely based on the apparent compatibility of the drug and carrier. Rigorous DFT analyses are necessary before any conclusions can be drawn regarding its therapeutic potential. The initial DFT analysis focused on reactivity descriptors to assess whether this system could be viable for cancer treatment. Table 1 summarizes the reactivity descriptors of CD, PROXI, and CD@PROXI.

Table 1 presents key chemical properties of CD, PROXI, and their conjugate CD@PROXI in both gas (g) and aqueous (aq) phases [29], shedding light on the potential of CD@PROXI as a targeted cancer treatment. The dipole moment of CD@PROXI is significantly higher in the aqueous phase (17.73 Debye) compared to its components in the gaseous phase form, indicating enhanced solubility and interactions in biological environments. This heightened polarity is beneficial for drug delivery systems designed to operate in physiological conditions. The hardness (η = 1.57 eV) shows that CD@PROXI is more reactive than PROXI (η = 2.34 eV) and CD (η = 1.68 eV), which supports its capability to interact effectively with biological targets. The softness (s) values further confirm this, with CD@PROXI exhibiting a relatively higher softness (s = 0.32 eV^−1^), indicating greater ease of deformation and higher reactivity [30].

The chemical potential (μ) of CD@PROXI in the aqueous phase (μ = −3.38 eV) is moderately negative, suggesting a balanced reactivity and thermodynamic stability, ideal for maintaining structural integrity while being active in biological systems. Importantly, the electrophilicity (ω) of CD@PROXI is notably high in the aqueous phase (ω = 3.64 eV), implying a strong tendency to accept electrons, which is crucial for engaging with nucleophilic sites in cancer cells [31]. Moreover, the presence of a C=N pH-responsive linkage in CD@PROXI enhances its functionality as a drug delivery system. This linkage can undergo protonation and structural changes in the slightly acidic environment of cancer cells, triggering the release of PROXI specifically in the target area.

Analysis of the data in Table 1 indicates that CD@PROXI shows strong potential as a candidate for targeted cancer treatment. This potential is attributed to its high dipole moment, optimal hardness and softness, balanced chemical potential, significant electrophilicity, and C=N pH-responsive linkage. These properties enable effective solubility, reactivity, and targeted release of the therapeutic agent in the acidic microenvironment of cancer cells, thereby maximizing treatment efficacy while minimizing off-target effects. Therefore, the analysis enables further exploration of CD@PROXI as a targeted treatment system through a series of DFT-based analyses, such as MO, DOS, ESP, IR spectra, C=N bond length, NCI, and ELF.

### 2.3. MO Analysis

**Figure 4:**
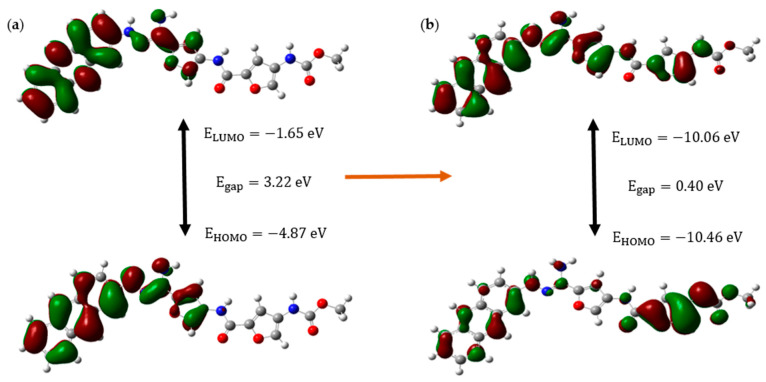
Molecular orbital analysis diagram of CD@PROXI in (**a**) neutral and (**b**) protonated states, respectively.

Figure 4 shows the HOMO-LUMO orbitals and their relevant energies of CD@PROXI in the neutral state, which represents the healthy cellular environment, and the protonated state, which represents the acidic cancerous environment.

The molecular orbital analysis of CD@PROXI, when depicted for both neutral and protonated states, shows significant electronic and structural changes that highlight its potential as a targeted cancer drug delivery system. When protonated, CD@PROXI experiences a significant decrease in its energy gap from 3.22 eV to 0.40 eV. This indicates a substantial increase in electronic reactivity, which is crucial for facilitating drug release in the acidic microenvironment of cancer cells. Moreover, the notable lowering of the LUMO and HOMO energy levels from −1.65 eV to −10.06 eV and −4.87 eV to −10.46 eV, respectively, further emphasizes the heightened electrophilic nature of the protonated state, enhancing its interaction with nucleophilic sites within cancer cells. These nucleophilic sites include the nitrogen and oxygen atoms in the bases of DNA and RNA, amino groups in lysine and arginine, thiol groups in cysteine, hydroxyl groups in serine, threonine, and tyrosine, as well as phosphate groups in lipids and carbohydrates. By selectively binding to these sites, CD@PROXI leverages the slightly acidic microenvironment characteristic of cancer cells to trigger the release of PROXI at the exact location where it is most effective. This protonation-induced shift in electronic properties, coupled with noticeable conformational changes observed in the molecular orbitals, supports an efficient and responsive release mechanism for PROXI. The data clearly demonstrate that CD@PROXI is designed to exploit the mildly acidic environment of cancer cells to activate its drug release mechanism, providing a refined and targeted approach to cancer therapy.

### 2.4. DOS Analysis

**Figure 5:**
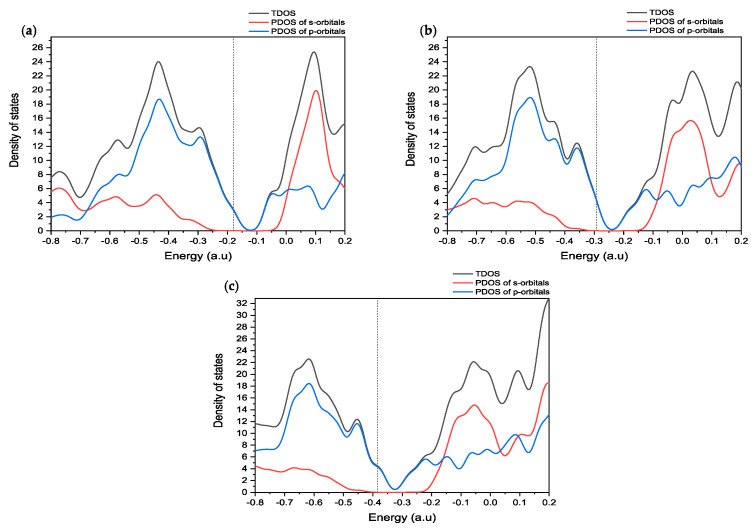
DOS plots for CD@PROXI in (**a**) neutral, (**b**) first protonation, and (**c**) second protonation states (dotted lines represent the Fermi level).

To understand the molecular orbital analysis of CD@PROXI as a targeted cancer drug delivery system in detail, a comprehensive study of the DOS is indispensable. The provided Figure 5 of DOS plots offers a meticulous examination of the electronic structure of CD@PROXI in both its neutral and protonated states, enabling a deeper understanding of its reactivity and interaction mechanisms.

The DOS plots presented in Figure 5 show a detailed study of the electronic properties of CD@PROXI, revealing critical insights into its behavior as a targeted cancer drug delivery system. DOS plots of the CD@PROXI system in neutral, first protonation, and second protonation states reveal critical insights into the electronic structure and bond dissociation favorability between CD and PROXI. In the neutral state, the DOS exhibits well-defined peaks, with significant contributions from p-orbitals near the Fermi level, indicating strong interactions between CD and PROXI. This suggests that bond dissociation is less favorable due to the stability of the electronic structure. Upon first protonation, a noticeable broadening of the peaks is observed, particularly in the s- and p-orbital contributions, reflecting increased electron redistribution and polarization. This alteration weakens the bond between CD and PROXI, making dissociation more favorable in acidic conditions of cancer cell environments. In the second protonation state, the DOS broadens further, with a significant increase in available electronic states, particularly in the conduction band, indicating a substantial weakening of the bond. The increased reactivity and electronic state availability suggest that bond dissociation becomes highly favorable in the second protonation state. Thus, protonation plays a critical role in modulating the bond strength, with higher protonation levels facilitating the release of PROXI in acidic environments, making CD@PROXI an effective pH-responsive system for targeted cancer therapy.

In summary, the molecular orbital and DOS analyses show that CD@PROXI is designed in a fashion to take advantage of the acidic surroundings of cancer cells. This enhances its reactivity and ability to release drugs in those cells, while it stays relatively inactive in the neutral environment of healthy cells. This selective reactivity is a crucial aspect of effective targeted cancer therapy.

### 2.5. ESP Analysis

**Figure 6:**
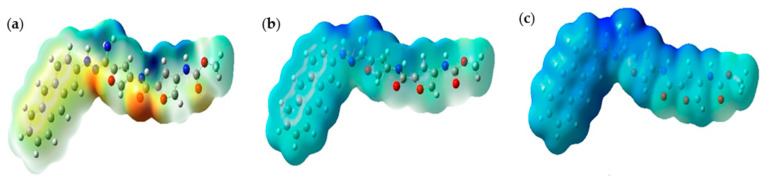
Electrostatic potential maps of CD@PROXI at (**a**) neutral, (**b**) first protonation, and (**c**) second protonation states (Blue, red, and yellow represent highly positive, highly negative, and moderate electrostatic potential regions).

Figure 6 below illustrates the ESP maps for CD@PROXI in neutral, first protonation, and second protonation states. These maps provide critical insights into CD@PROXI’s electronic distribution and reactivity, underpinning its design as a targeted cancer drug delivery system. In these maps, blue regions indicate areas of high positive electrostatic potential (electrophilic sites), while red regions indicate areas of high negative electrostatic potential (nucleophilic sites).

In the neutral state, the balanced distribution of electrostatic potential indicates moderate reactivity, suitable for stability in the neutral pH environment of healthy cells. Upon first protonation, the molecule becomes more electrophilic, with an increase in positive electrostatic potential regions, suggesting enhanced reactivity in slightly acidic environments like those found in cancer cells. The second protonation state shows an intense blue region near the C=N bond linkage, indicating a highly electrophilic and reactive site. This region’s pronounced electron deficiency makes it a focal point for interactions with nucleophilic sites, such as DNA, RNA, and proteins, within the acidic microenvironment of cancer cells. The high positive potential near the C=N bond suggests it is a preferred binding site for the therapeutic agent, facilitating efficient drug release. This selective reactivity ensures that CD@PROXI remains stable and less reactive in healthy cells while becoming highly reactive in cancer cells, leading to targeted drug release. The design of CD@PROXI thus effectively balances stability and reactivity, ensuring selective activation and drug release in cancerous tissues, which is crucial for effective and efficient cancer therapy.

### 2.6. IR Spectroscopy and C=N Bond Length

**Figure 7:**
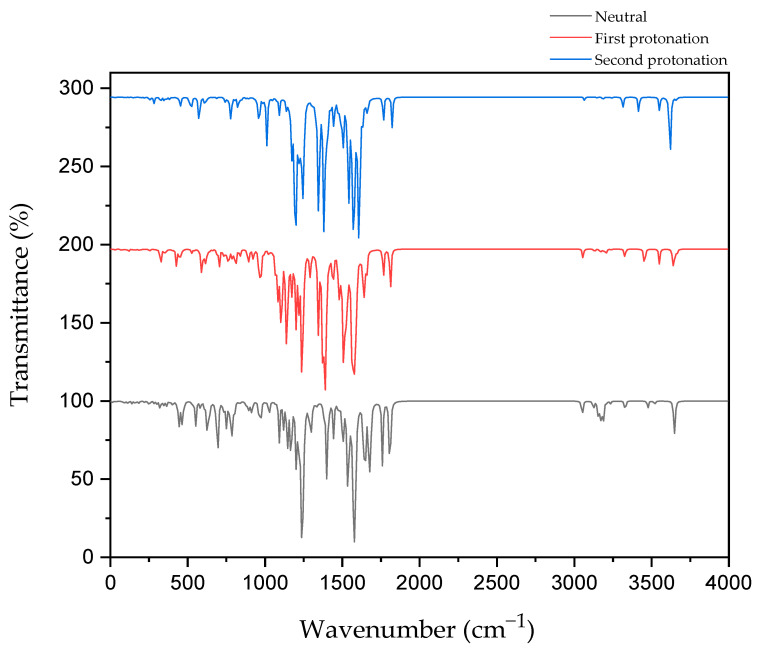
IR spectrum of CD@PROXI in neutral, first protonation, and second protonation states.

Figure 7 shows the IR spectroscopy of the C=N bond in CD@PROXI in neutral, first protonation, and second protonation states.

The IR spectroscopy data for CD@PROXI across different protonation states provides critical insights into the behavior of the C=N bond, which acts as the bridge between the CD and PROXI. C=N stretch typically falls within the range of 1640–1690 cm^−1^ for aliphatic C=N. In neutral conditions, the C=N bond shows its stretch at ~1650 cm^−1^. Upon first protonation, the bond stretch shifts to ~1640 cm^−1^. After the second protonation, the stretch further shifts to ~1610 cm^−1^. These IR vibration shifts reflect protonation-induced weakening due to electronic effects, reducing the vibrational strength and rendering the C=N bond more susceptible to dissociation. The IR data are relevant to the C=N stretch and were further ensured by the increase in the C=N bond length, wherein the neutral state, it showed a bond length of 1.288 Å. In the first and second protonation states, it showed further stretching with bond lengths increasing to 1.310 Å and 1.324 Å, respectively.

This weakening is further supported by the electrostatic potential maps of CD@PROXI, which show significant changes around the C=N bond region upon protonation. In the neutral state, the electrostatic potential maps exhibit balanced green regions, indicating neutral charge distribution. With protonation, blue regions emerge around the C=N bond, signifying an increase in positive electrostatic potential. This change highlights the bond’s increased reactivity under acidic conditions typical of cancer cell microenvironments. The protonation-induced dissociation of the C=N bond leads to the cleavage of the bridge between the CD and PROXI, reforming the original molecules. This targeted dissociation allows PROXI to be selectively released in the acidic environment of cancer cells, where it interacts directly with cancer cells to inhibit growth or induce apoptosis.

Importantly, the CD acts as a stable nanocarrier throughout this process. The stability of the CD is evident in the minimal spectral changes of other bonds, including the aromatic C=C stretching vibrations (~1400–1600 cm⁻^1^). These vibrations remain unaffected by protonation, confirming the robustness of the aromatic framework of the CD. This stability ensures that the CD does not interact with cancer cells and remains biologically inert, facilitating safe excretion from the body after delivering its therapeutic payload.

By leveraging the protonation-sensitive dissociation of the C=N bond while maintaining the stability of the CD, CD@PROXI ensures the selective release of PROXI in acidic tumor environments. This mechanism not only enhances the therapeutic efficacy of PROXI but also minimizes systemic toxicity by sparing healthy cells. The combination of insights from IR spectroscopy and ESP maps highlights the precision of CD@PROXI’s design as a pH-responsive, stable nanocarrier for targeted cancer therapy.

### 2.7. NCI Analysis

**Figure 8:**
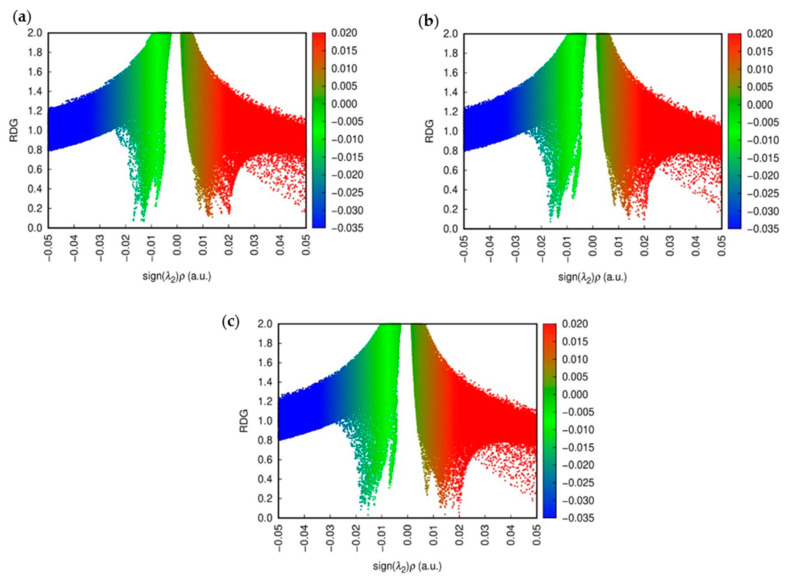
NCI plots of CD@PROXI in (**a**) neutral, (**b**) first protonation, and (**c**) second protonation states.

NCI plots were utilized to visualize and analyze non-covalent interactions such as hydrogen bonding, van der Waals forces, and π-π stacking within molecular systems. These plots highlight interactions through color-coded isosurfaces: blue for strong attractive interactions, green for weaker van der Waals interactions, and red for repulsive interactions. By analyzing the reduced density gradient (RDG) of electron density, NCI plots identify regions of non-covalent interactions, aiding in distinguishing attractive from repulsive forces. This visualization technique is crucial for understanding molecular recognition, binding affinities, and structural integrity in biological molecules, materials science, and molecular engineering. Figure 8 shows the NCI colored plots for CD@PROXI in neutral, first protonation, and second protonation states [32].

The above analysis shows how the CD@PROXI system behaves in various protonation states, particularly in acidic cancer cell environments. In the neutral state, the NCI plot indicates stable non-covalent interactions, with hydrogen bonds and van der Waals forces contributing to a robust bond, making dissociation less favorable. Upon first protonation, the system shows a slight increase in green and red regions, which corresponds to van der Waals interactions and repulsion, signaling a weakening of attractive forces, which makes bond dissociation more favorable. In the second protonation state, there is a significant rise in repulsive interactions alongside weak van der Waals forces, indicating a highly destabilized system where bond dissociation is strongly favored. This analysis underscores the role of protonation in modulating the stability of the CD@PROXI complex, enhancing its suitability as a pH-responsive drug delivery system for targeted cancer therapy, with increased bond dissociation in an acidic cancer cell environment.

### 2.8. ELF Analysis

**Figure 9:**
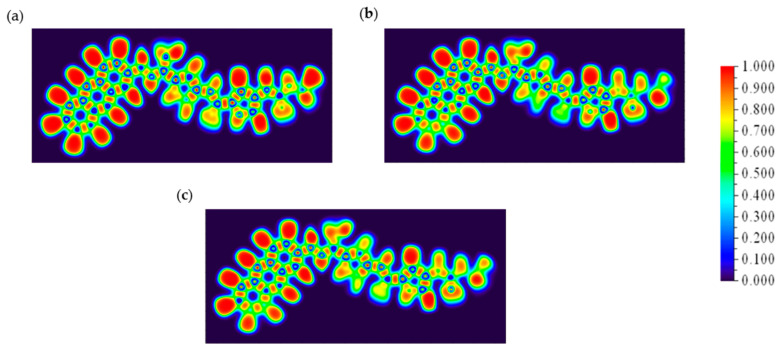
ELF plots of CD@PROXI in (**a**) neutral, (**b**) first protonation, and (**c**) second protonation states.

The provided ELF plots for the CD@PROXI system in Figure 9 illustrate the electronic structure in different states: neutral, first protonation, and second protonation. These plots are crucial for understanding how protonation affects electron localization and, consequently, the favorability of PROXI release specifically in cancer cells.

In the neutral state, the ELF plot shows high values concentrated around specific areas of the molecule. These regions of high ELF values indicate strong electron pair localization, which corresponds to stable covalent bonds and lone pairs. This localization suggests that in the neutral state, the molecule is relatively stable and electron-dense, ensuring that PROXI remains securely bound within the CD@PROXI complex during transport through the body and preventing premature release.

Upon the first protonation, the ELF distribution changes noticeably. The previously concentrated high ELF regions become more dispersed. The addition of a proton introduces a positive charge, affecting the electron density around these high electron localization areas. This shift suggests a destabilization of electron pairs in these regions, indicating that protonation begins to weaken the binding of PROXI within the CD complex, making it more likely to be released. This initial protonation step appears to prime the molecule for dissociation by altering its electronic structure.

Further protonation leads to a slight regain of localization in delocalized regions. This may indicate the stabilization of PROXI as a separate fragment upon further protonation, suggesting that bond weakening between CD and PROXI within CD@PROXI. Such changes are highly favorable for the complete dissociation of PROXI from the CD complex [33]. The pronounced alteration in electron localization suggests that the molecule is becoming increasingly unstable, thus facilitating its release in an environment with higher proton concentration, such as the acidic environment characteristic of cancer cells.

Since cancer cells typically present a more acidic environment than normal cells, the observed changes in ELF strongly support the hypothesis that CD@PROXI is tailored to release PROXI specifically in proximity to cancer cells. The protonation-induced changes in electron localization favor the targeted release, thereby enhancing therapeutic efficacy while minimizing effects on normal cells. Thus, ELF analysis supports the favorability of PROXI release at cancer cells, validating the design of the CD@PROXI targeted treatment system as an effective means of delivering cancer therapy.

## 3. Computational Methodology

### 3.1. Initial Optimization

The CD optimization was performed using Gaussian 09W software by employing the B3LYP functional with empirical dispersion correction (D3BJ) and the 6-311G (d, p) basis set to obtain its stable configuration, followed by a molecular orbital (MO) analysis to assess its validity as a drug carrier [34]. Given the complexity of CD@PROXI consisting of first-row elements H, C, N, and O, the B3LYP functional offers a good balance between computational cost and accuracy in capturing the electronic structure of CD@PROXI, while D3BJ would account for crucial non-covalent interactions, such as van der Waals interactions within CD@PROXI, by significantly enhancing the accuracy of B3LYP [35,36,37,38]. On the other hand, the 6-311G triple-zeta split-valence basis set provides an effective description of the electronic structure of CD@PROXI with additional d and p polarization functions, which improve accuracy in capturing electronic properties, particularly HOMO-LUMO energies and charge distribution, which is useful for studying electrostatic potential and electron localization function (ELF) maps [39]. Then PROXI was optimized using the same computational parameters to obtain its most stable configuration, and an ESP map analysis was performed to validate its potential as an anticancer agent.

### 3.2. pH-Sensitive Conjugation and Reactivity Descriptors Calculation

After verifying the individual components, the CD and PROXI were conjugated to form the CD@PROXI system via a pH-sensitive C=N linkage. Then the CD@PROXY structure was verified as suitable to develop as a drug system by confirming its high solubility and validity as a drug system. For that, an MO analysis was performed on CD@PROXY. Under MO analysis, the HOMO (highest occupied molecular orbital) and LUMO (lowest unoccupied molecular orbital) of CD@PROXI were generated in the gaseous phase and aqueous phase. By using the energies *E_HOMO_* and *E_LUMO_* related to HOMO and LUMO orbitals, several reactivity descriptors like chemical hardness, chemical softness, chemical potential, and electrophilicity were derived. Equations (1)–(4) below represent chemical hardness, chemical softness, chemical potential, and electrophilicity, respectively.

(1) $$\eta =\frac{\left(IP-EA\right)}{2}$$

(2) $$s=\frac{1}{2\eta }$$

(3) $$\mu =-\frac{\left(IP+EA\right)}{2}$$

(4) $$\omega =\frac{\mu^{2}}{2\eta }$$

where η is hardness, s is softness, μ is chemical potential, and ω is electrophilicity. The *IP* and *EA* were taken as *IP* = −*E_HOMO_* and *EA* = −*E_LUMO_* [40,41,42,43,44]. Along with the above parameters, the dipole moment of CD@PROXY was also considered in both phases to verify its solubility.

### 3.3. Comprehensive DFT-Based Analyses

After validity and solubility verification, the CD@PROXI structure underwent a comprehensive series of DFT-based analyses, such as finding the energy gap (E_gap_) using MO analysis, density of states (DOSs), ESP, IR spectroscopy, non-covalent interactions (NCIs), and ELF. The plots related to DOS, NCI, and ELF were generated using Multiwfn 3.8 software, while ESP maps, IR spectroscopy, and MO diagrams were generated using the Gaussian 09W software. The data relevant to DOS and IR spectroscopy were extracted from Multiwfn 3.8 and Gaussian software to Origin 2018, and they were plotted. Data relevant to the NCI plot was obtained from Multiwfn 3.8 software and plotted using gnuplot 6.0. Since CD@PROXY has the pH-sensitive C=N bond, it can protonate twice and cleave the bond in low pH conditions. Therefore, each analysis was performed for the neutral, first protonation, and second protonation states of CD@PROXI. The neutral state of CD@PROXI represents its structure in a healthy cell environment, while the two protonation states represent the acidic cancer cell environment. The neutral structure is the initially built CD@PROXI structure, while the first protonation state was obtained by assigning a ‘+’ charge to the neutral structure, and the second protonation state was obtained by assigning two ‘+’ charges to the neutral structure. Then the structures were subjected to the above-mentioned analyses, which provided detailed insights into the electronic properties, stability, and reactivity of the CD@PROXI system, confirming its potential as an effective and selective targeted drug delivery system for cancer therapy. ChatGPT (OpenAI) was used as a writing assistant to refine the manuscript’s language and improve clarity.

## 4. Conclusions

DFT simulations in this study evaluated the CD (carbon dot) functionalized with PROXI (proximicin-A) as a targeted drug delivery system for cancer treatment. The CD’s structure was optimized, confirming its stability as a drug carrier, while PROXI’s potential interaction sites and charge distribution were analyzed using ESP maps. Comprehensive DFT-based analyses of the CD@PROXI conjugate, including MO, DOS, ESP, IR spectroscopy, NCI, and ELF, demonstrated that CD@PROXI remains stable in neutral pH environments but dissociates specifically in acidic cancer cell environments by considering the impact of different protonation states, confirming that CD@PROXI is effective under acidic conditions of cancer cells. The MO and DOS analyses revealed increased reactivity in acidic conditions, while ESP analysis pinpointed the region of increased reactivity, and IR spectroscopy identified the C=N bond between CD and PROXI as a key factor in bond dissociation. NCI and ELF analyses confirmed that CD@PROXI possesses the necessary stability, reactivity, and electronic properties for selective cancer treatment. This targeted delivery system can deliver PROXI specifically to cancer cells, minimizing damage to healthy cells and reducing side effects, indicating its potential for further development and clinical application.

## Figures and Tables

**Table 1:** Reactivity descriptors of CD, PROXI, and CD@PROXI.

	Dipole Moment (Debye)	Hardness (η)/eV	Softness (s)/eV^−1^	Chemical Potential (μ)/eV	Electrophilicity (ω)/eV
CD (g)	4.23	1.68	0.30	−3.19	3.03
PROXI (g)	12.81	2.34	0.21	−3.86	3.18
CD@PROXI (g)	10.84	1.61	0.31	−3.26	3.30
CD@PROXI (aq)	17.73	1.57	0.32	−3.38	3.64

## Data Availability

The original contributions presented in the study are included in the article; further inquiries can be directed to the corresponding author.
